# Supplementary material for: In Vitro Anthelmintic Evaluation of Gliricidia sepium, Leucaena leucocephala, and Pithecellobium dulce: Fingerprint Analysis of Extracts by UHPLC-Orbitrap Mass Spectrometry
Source: Molecules. 2020 Jun 30;25(13):3002. doi: 10.3390/molecules25133002 (PMC7412154; doi:10.3390/molecules25133002)
Supplement: Supplementary file 1 [file molecules-25-03002-s001.pdf]

Supplementary material

In vitro Anthelmintic Evaluation of *Gliricidia sepium*, *Leucaena leucocephala*, and *Pithecellobium dulce*: Fingerprint Analysis of Extracts by UHPLC-Orbitrap Mass Spectrometry

Néstor Romero[a]*, Carlos Areche[b], Andrés Cubides-Cárdenas[c], Natalia Escobar[d], Olimpo García-Beltrán[e], Mario Simirgiotis[f], Ángel Céspedes[a]

[a]*Departamento de Sanidad Animal, Facultad de Medicina Veterinaria y Zootecnia, Universidad del Tolima, Ibagué, Colombia*; aecesped@ut.edu.co (A.C.)

[b]*Departamento de Química, Facultad de Ciencias, Universidad de Chile, Las Palmeras 3425, Nuñoa, Santiago 7800024, Chile*; areche@uchile.cl (C.A.)

[c]*Grupo de Investigación e Innovación en Salud y Bienestar Animal, Laboratorio de Salud Animal, Centro de Investigación Tibaitatá, Agrosavia, Mosquera, Colombia*; jcubides@agrosavia.co (A.C.-C)

[d]*Facultad de Ciencias Agropecuarias, Universidad de Cundinamarca, Fusagasugá, Colombia;* nataliaescobar@ucundinamarca.edu.co (N.E.)

[e]*Facultad de Ciencias Naturales y Matemáticas, Universidad de Ibagué, Carrera 22 Calle 67, Ibagué 730002, Colombia*; jose.garcia@unibague.edu.co (O.G-B.),

[f]*Instituto de Farmacia, Universidad Austral de Chile, Valdivia 5090000, Chile*; mario.simirgiotis@uach.cl (M.S.)

***Correspondence:** njromeroj@ut.edu.co (N.R)

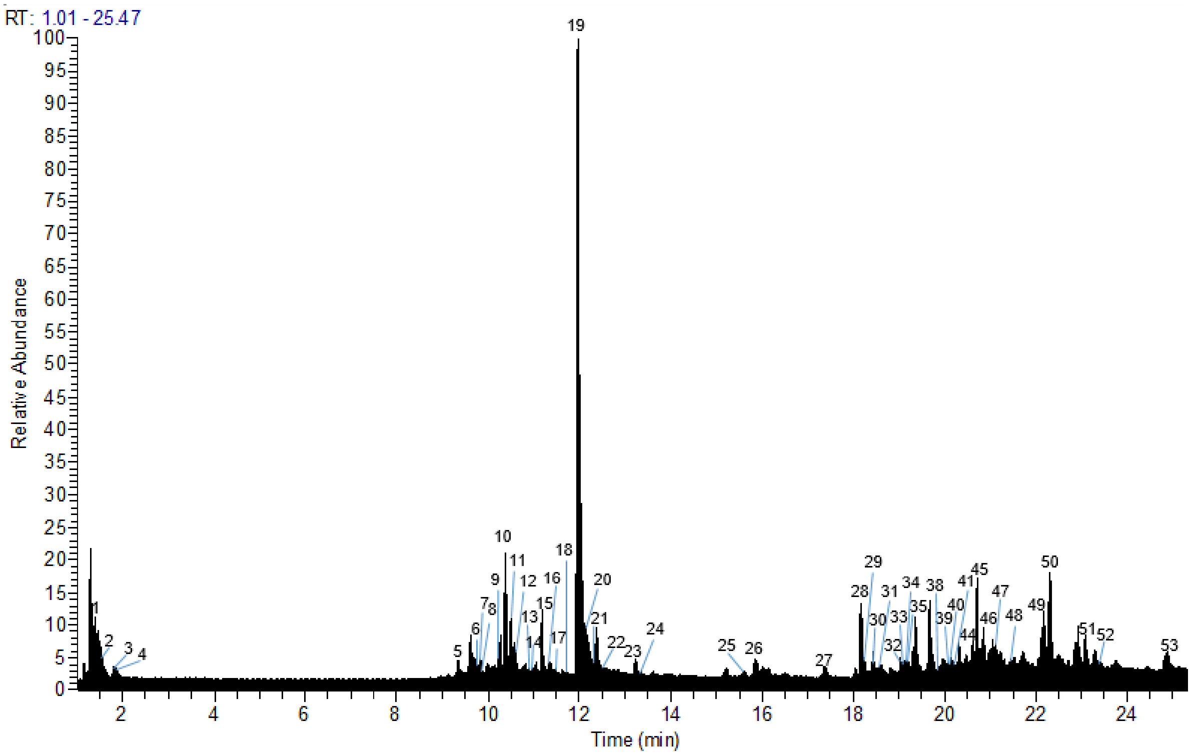

Figure S1. UHPLC TIC (total ion current) chromatogram of *Gliricidia sepium* ethanolic extracts.

Table S1. Tentative identification of compounds in the ethanolic extract of *Gliricidia sepium* by UHPLC-Q/Orbitrap/MS/ MS

Peak	Tentative identification	[M-H]$^-$ ions	Retention time (min.)	Theoretical mass (*m/z*)	Measured mass (*m/z*)	Accuracy (ppm)	Other Ions (m/z)*	UV vis (nm)
1	Tetrahydroxypentanal (aldopentose)	$C_5H_9O_5^-$	1.38	149.0444	149.0449	2.7	131.0342	265
2	Tetrahydroxypentanal (aldopentose) Isomer	$C_5H_9O_5^-$	1.52	149.0444	149.0449	3.2	131,0344	334
3	Uridine	$C_9H_{11}O_6N_2^-$	1.79	243.0612	243.0625	5.3	157.0307	205, 263
4	Glutamic acid	$C_5H_8NO_4^-$	1.88	146.0452	146.0448	2.9	102.0553, 128.0345 129.0186	260
5	Hexahydroxytrioxoicosanoic acid	$C_{20}H_{33}O_{11}^-$	9.38	449.2017	449.2030	2.7	NF.	268, 320, 350
6	Tetrahydroxytetraoxoicosanoic acid	$C_{20}H_{31}O_{10}^-$	9.75	431.1912	431.1925	3.1	N.F.	244, 265, 344, 378
7	Trihydroxypentaoxooctadecanoic acid	$C_{18}H_{25}O_{10}^-$	9.82	401.1442	401.1455	3.2	N.F.	260
8	unknown	$C_{24}H_{21}O_8^-$	9.84	437.1231	437.1223	-1.9	-	244, 261
9	Phenyllactic acid-2-O-glucoside	$C_{15}H_{19}O_8^-$	10.21	327.1074	327.1087	3.9	121.0651, 165.0556 (phenyllactic acid)	246, 265
10	Apigenin-di-C-dihexose –O-deoxyhexose	$C_{33}H_{39}O_{19}^-$	10.38	739.2091	739.2073	2.0	163.0608, 593.1512 (apigenin dihexosa)	219, 267, 345
11	p-coumaroyl hexose	$C_{15}H_{17}O_8^-$	10.54	325.0918	325.0931	3.9	119.0494, 163.0394 (coumaric acid)	275
12	Apigenin-di-C-dihexose –O-deoxyhexose isomer	$C_{33}H_{39}O_{19}^-$	10.63	739.2091	739.2074	2.2	163.0607, 593.1510 (apigenin dihexosa)	265, 341
13	Rutin	$C_{27}H_{29}O_{16}^-$	10.90	609.1461	609.1459	0,3	137.0237, 149.0238,	260, 284
14	Caffeoyl hexoside	$C_{15}H_{17}O_9^-$	10.98	341.0867	341.0880	3.6	163.0607, 135.0445, 179.0345 (caffeic acid)	270
15	Trihydroxyanthraquinone-O-methylgluconate-glucoside	$C_{27}H_{29}O_{15}^-$	11.22	593.1501	593.1508	1.1	219.0877, 233.1026, 251.1118, 255,0297 (trihydroxyanthraquinone)	265, 292, 346

16	Trihydroxyanthraquinone- O-methylgluconate-deoxymethylgluconic	$C_{28}H_{31}O_{16}^{-}$	11.35	623.1618	623.1611	1.1	121.0287, 135.0080 249.0961	265, 338
17	Trihydroxyanthraquinone-O-methylgluconate	$C_{21}H_{19}O_{11}^{-}$	11.49	447.0922	447.0936	3.1	115.0393, 117,0550, 119.0343, 131.0344, 135.0658, 147.0657, 255.0299	265, 321
18	Trihydroxyanthraquinone-O-methylgluconate isomer	$C_{21}H_{19}O_{11}^{-}$	11.75	447.0922	447.0936	3.1	115.0392, 119.0344, 147.0655	274
19	Dihydro-p-coumaric acid isomer	$C_9H_9O_3^{-}$	11.98	165.0546	165.0552	3.3	121.0651	213, 273
20	Ázelaic acid	$C_9H_{15}O_4^{-}$	12.18	187.0976	187.0972	4.0	121.0651, 123.0808, 139,0757, 141.0915, 143.1071, 169.0867, 171.1020	270
21	p-coumaric acid	$C_9H_7O_3^{-}$	12.37	163.0390	163.0396	3.6	117.0338, 119.0496, 121,0286, 135.0446, 137.0238	238, 276, 325
22	Dihydroxydioxooctadecanoic acid	$C_{18}H_{31}O_6^{-}$	12.53	343.2115	343.2128	3.7	NF	257
23	N-Carbobenzyloxy-L-isoleucine	$C_{14}H_{18}O_4N^{-}$	13.26	264.1230	264.1242	4.4	128,0346, 130.0503, 130.0866 (isoleucine), 150.0557, 152.0347, 156.0659, 158.0816, 220.1340, 246.1131	277, 307
.24	Apigenin-7-O- glucoside	$C_{21}H_{19}O_{10}^{-}$	13.35	431.0973	431.0985	2.9	117.0549, 161.0449, 253.0504 (apigenin aglycon)	275
25	Leu/dihydro-p-coumaric acid	$C_{15}H_{20}NO_4^{-}$	15.59	278.1387	278.1398	4.1	119.0495, 121.0651 130.0866, 165.0552 (dihydro-p-coumaric acid)	284
26	Trihydroxy-octadecadienoic acid	$C_{18}H_{31}O_5^{-}$	15.85	327.2166	327.2178	3.6	155.0707, 159,1020, 169.0865, 185,0810, 187.0968, 197,1181, 309.2076	284
27	Phe /Dihydro-p-coumaric acid	$C_{18}H_{18}NO_4^{-}$	17.38	312.1230	312.1244	4.2	119.0495, 121.0288,147.0445, 163.0394, 164.0712 (phenylalanine), 174.0555, 268.1343	284, 329
28	Dihydroxydodecadienoic acid	$C_{12}H_{19}O_4^{-}$	18.18	227.1278	227.1287	3.9	165,1279, 183.1388,191.1073, 209.1179	283
29	Tianshic Acid	$C_{18}H_{33}O_5^{-}$	18.26	329.2323	329.2336	4.1	167.1071, 171,1020,185.1180, 195.1021,197.1179, 213.1129,	283

							215.1288, 227.1287,243.1234, 311.2227	
30	Dihydroxydodecadienoic acid isomer	$C_{12}H_{19}O_4^-$	18.41	227.1278	227.1287	3.9	165.1280, 183.1386, 209.1180	283
31	Trihydroxyoctadecadienoic acid isomer	$C_{18}H_{31}O_5^-$	18.53	327.2166	327.2179	4.0	155.0706, 157.0868, 169.0863, 185.0814, 187.0972, 197.1179, 217.1076	283
32	7-Hydroxy-4'-methoxyisoflavone (Formononetin)	$C_{16}H_{11}O_4^-$	19.03	267.0652	267.0664	4.6	195.0445, 223.0394	283
33	Tianshic acid isomer	$C_{18}H_{33}O_5^-$	19.11	329.2323	329.2336	4.2	127.1121, 141.0919, 155.1072, 171.1020, 185.1180, 197.1183, 199.1336, 213.1128, 215.1288, 227.1287, 243.1237, 293.2125, 311.2229	221, 274
34	Tianshic acid derivative	$C_{18}H_{29}O_4^-$	19.15	309.2060	309.2073	4.1	155.0708, 169,0865, 171.1021, 185,1180, 197.1184, 273,1860, 291,1974	222, 274
35	Tridecadienedioic acid	$C_{13}H_{19}O_4^-$	19.18	239.1278	239.1288	4.0	N.F.	275
36	Unknow	-	19.21	-	939.4911	-	-	276
37	Unknow	-	19.36	-	941.5068	-	-	282
38	Dihydrocapsiate	$C_{18}H_{27}O_4^-$	19.84	307.1904	307.1917	4.3	125.0965, 127.0758, 137.0966, 139.1121, 155.0707, 167.0709, 171.1023, 179.1072, 183.1022, 195.1023,209.1183, 247.1703, 289.1811	282
39	Tianshic acid derivative	$C_{18}H_{29}O_4^-$	20.06	309.2060	309.2074	4.4	125.0965, 137.0964, 153.0917, 155.0707, 171.1020, 185.1179, 197.1181, 213.1130	282
40	Unknow	$C_{29}H_{51}O_{15}N_1$	20.08	-	779.4565	-	-	282
41	Hydroxydioxoheptadecenoic acid	$C_{17}H_{27}O_5^-$	20.15	311.1853	311.1866	4.0	153.1279, 171,1021, 193.0867, 195.1025, 197.1180, 211.1342, 237.1129, 239.1287, 267.1236, 267.1965, 293.1755	282
42	Unknow	$C_{30}H_{53}O_{15}N_1^-$	20.31	-	793.4355	-	-	282
43	Unknow	$C_{27}H_{39}O_7^-$	20.51	475.2690	475.2704	-	-	281

44	Dihydroxyoctadecadienoic acid	$C_{18}H_{31}O_4^-$	20.63	311.2217	311.2229	4.0	157.0865, 173.1176, 183.1380, 211.1337, 223.1339, 239.1288, 293.2123	281
45	Phenethyl Butyrate	$C_{12}H_{15}O_2^-$	20.73	191.1067	191.1074	4.0	121.0650, 175.0762,	282
46	p-coumaric acid deriative	$C_9H_{11}O_2^-$	20.84	151.0754	151.0759	3.5	119.0502, 121,0651	281
47	Tianshic acid derivative	$C_{18}H_{29}O_4^-$	21.13	309.2060	309.2074	4.4	153.1278, 167.1073, 169.1228, 179.1437, 181.1231, 195.1386, 197.1180, 211.1336, 213.1129, 263.2017, 281.1759, 291.1965	281
48	Marrubiin	$C_{20}H_{27}O_4^-$	21.46	331.1904	331.1917	4.0	193.1591, 247.1340, 259.2071, 287.2014	278
49	Hydroxyoctadecatrienoic acid	$C_{18}H_{29}O_3^-$	22.16	293.2111	293.2123	4.1	231.2116, 235.1701, 275.2015, 277.1805	248, 275
50	Hydroxyoctadecatrienoic acid isomer	$C_{18}H_{29}O_3^-$	22.30	293.2111	293.2123	4.1	231.2123, 275.2015	248, 275
51	Hydroxylinoleic acid	$C_{18}H_{31}O_3^-$	23.08	295.2268	295.2280	4.1	277.2171, 195.1386	275
52	Pentadecatetraenoic acid	$C_{15}H_{21}O_2^-$	23.39	233.1536	233.1546	4.2	-	275
53	Unknow	$C_{30}H_{41}O_{11}^-$	24.88	577.2649	577.2686	-6.4	-	275

* Daughter ions

Compounds associated with anthelmintic activity in Gliricidia sepium

Glycosylated flavonoids. Four glycosylated flavonoids (peaks 10, 12, 13 and 24) were identified in the ethanolic extract using UHPLC-Q/Orbitrap/MS/MS. Peak 10 with a [M−H]−ion *m/z* 739.2073 and daughter ions at *m/z* 163.0605 (deoxyhexose), 593.1502 (apigenin dihexose), 431.0981 and 285.0402 were designated as apigenin-di-C-dihexose-O-deoxyhexose [1]. Peak 12 with a [M−H]−ion *m/z* 739.2074 and daughter ions at m/z 163.0607 (deoxyhexose) and 593.1510 (apigenin dihexose) were called apigenin-di-C-dihexose–O-deoxihexosa isomer [1]. Peak 13 with a [M−H]−ion *m/z* 609.1459 and daughter ions at m/z 137.0237, 149.0238 were identified as rutin [2]. Peak 24 with a [M−H]−ion *m/z* 431.0985 and daughter ions at *m/z* 253.504 (apigenin aglycone) and 161.0449 (hexoside), were identified as apigenin-7-O-glucoside [3].

Phenylpropanoids other than flavonoids. Seven phenylpropanoids other than flavonoids corresponding to peaks 11, 14, 19, 21, 25, 27 and 46 were identified using UHPLC-Q/Orbitrap/MS/MS. Peak 11 with a [M−H]−ion *m/z* 325.0931 and the daughter ions at m/z 163.0394 (coumaric acid) and 119.0494 were identified as p-cumaroyl hexose [4]. Peak 14 with a [M−H]−ion *m/z* 341.0880, daughter ions at *m/z* 179.0345 (caffeic acid), 163.0607 and 135.0445 were identified as caffeoyl hexoside [5]. Peak 19 with a [M−H]−ion *m/z* 165.0552 and daughter ion 121.0651, were identified as isomer of dihydro-*p*-coumaric acid [6]. Peak 21 with a [M−H]− ion *m/z* 163.0396 and daughter ions at *m/z* 119.0494, 121.0286, 135.0446 and 137.0238 were identified as *p*-coumaric acid [7,8] also [9] identified the same acid in *G. sepium*. Peak 25 with a [M−H]−ion *m/z* 278.1398 and daughter ions at *m/z* 165.0552 (dihydro *p*-coumaric acid) and 130.0866 (leucine), were identified as leucine/dihydro-*p*-coumaric acid. Peak 27 with a [M−H]−ion *m/z* 312,1244 and daughter ions at *m/z* 119,0494, 121,0288, 147,0445, 163,0394 and 164,0712 (phenylalanine), were identified as phenylalanine/dihydro-*p*-cumaric acid. Peak 46 with a [M− H]−ion *m/z* 151.0759 and daughter ions at *m/z* 119.0502, 121.0651 and 133.0652 were identified as derived from *p*-coumaric acid.

Methoxyphenols. One phenol was identified using UHPLC-Q/Orbitrap/MS/MS. Peak 38 with a [M−H]−ion *m/z* 307.1917 and daughter ions at *m/z* 125.0965, 127.0758, 137.0966, 139.1121, 155.0707, 167.0709 171.1023, 179.1072 183.1022, 195.1023, 209.1183, 247.1703 and 289.1811 were identified as dihydrocapsiate [10].

Anthraquinonic glycosides. Four different anthraquinonic glycosides (peaks 15, 16, 17 and 18) were identified using UHPLC-Q/Orbitrap/MS/MS. Peak 15 with a [M−H]−ion *m/z* 593.1508 and daughter ions at *m/z* 121.0287, 219.0877, 233.1026, 251,1118 and 255,0297 (trihydroxi- anthraquinone), were

identified as trihydroxy-anthraquinone-O-methylgluconate-glycoside [11]. Peak 16 with a [M−H]−ion *m/z* 623.1611 and daughter ions at *m/z* 593.1508, 249.0961, 121.0287 and 135.0080 were identified as trihydroxy-anthraquinone-O-methylgluconate-deoxymethylgluconic. Peak 17 with a [M−H]−ion *m/z* 447.0936 and daughter ion at *m/z* 255.0299 (trihydroxy-anthraquinone) were identified as trihydroxy-anthraquinone-O-methylgluconate [11]. Peak 18 with [M−H]−ion *m/z* 447.0936 and daughter ions at *m/z* 115.0392, 119.0344 and 147.0655 were identified as a trihydroxy-anthraquinone-O-methylgluconate isomer.

Amino acids. Two amino acids (peaks 4 and 23) were identified in the ethanolic extract using UHPLC-Q/Orbitrap/MS/MS. Peak 4 with a [M−H]−ion *m/z* 146.0448 and daughter ions at *m/z* 102.0553, 128.0345, 129.0186 were identified as glutamic acid. Peak 23 with a [M− H]−ion *m/z* 264.1242 and daughter ions at *m/z* 130.0866 (isoleucine) and 128.0346, 130.0503, 150.0557, 152.0710, 156.0659, 158.0816, 220.1340 and 246.1131 were identified as N-Carbobenzyloxy-L-isoleucine.

Glycosylated phenolic acids. One glycosylated phenolic acid was identified using UHPLC-Q/Orbitrap/MS/MS. Peak 9 with a [M−H]$^-$ ion *m/z* 327.1087 and characteristic daughter ions at *m/z* 165.0556 (phenylactic acid), 121.0651, 147.0446 were identified as phenyllactic acid-2-O -glucoside [4].

Fatty acids. One fatty acid was identified using UHPLC-Q/Orbitrap/MS/MS. Peak 20 with a [M− H]$^-$ ion *m/z* 187.0972 and daughter ions at *m/z* 121.0651, 123.0808, 139.0757, 141.0915, 143.1071, 169.0867 and 171.1020 were identified as azelaic acid [12].

Figure S2 shows the UHPLC total ion current chromatogram and Table S2 shows all the compounds detected and identified by UHPLC-Q/Orbitrap/MS/MS in *L. leucocephala*.

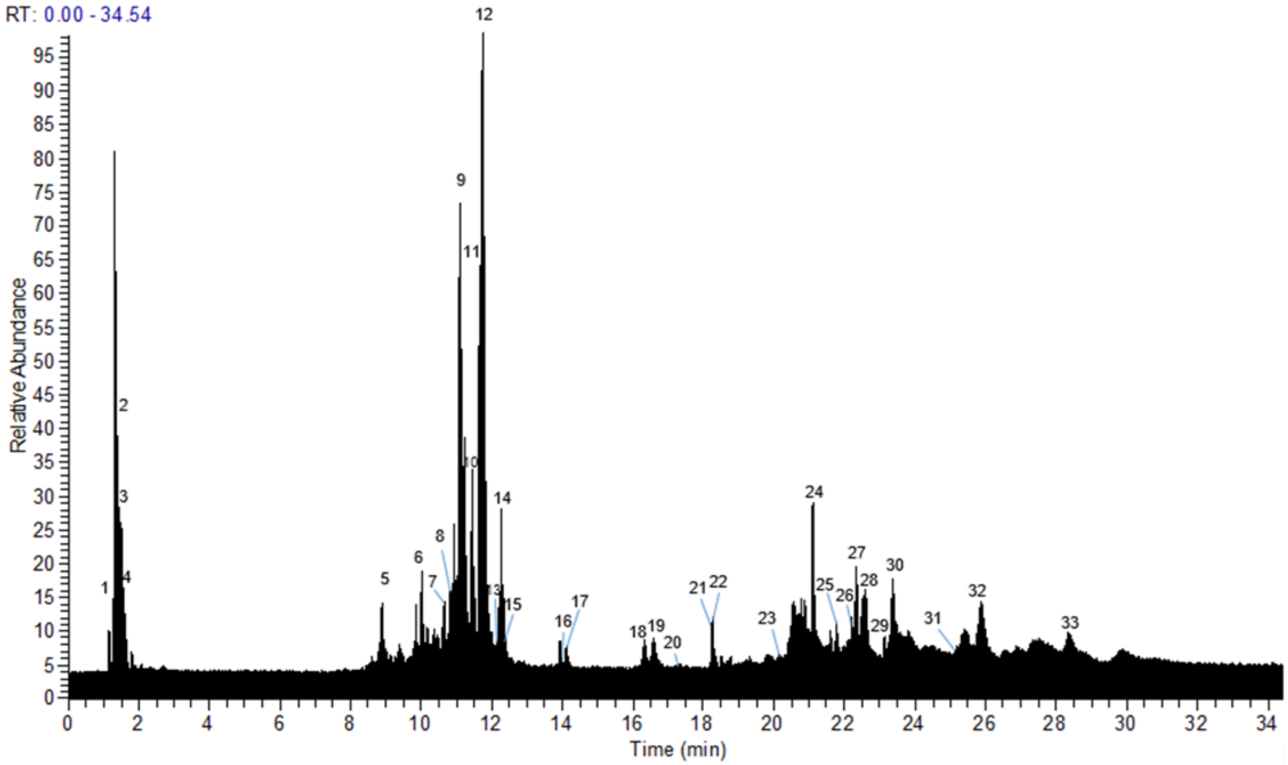

Figure S2. UHPLC TIC Chromatogram *Leucaena leucocephala* ethanolic extract

Table S2. Tentative identification of compounds of the ethanolic extract of *Leucaena leucocephala* by UHPLC-Q/Orbitrap/ MS/MS

Peak	Tentative identificaction	[M-H]$^-$ ions	Retention time (min.)	Theoretical mass (*m/z*)	Measured mass (*m/z*)	Accuracy (ppm)	Other ions (*m/z*)*	UV vis (nm)
1	Gluconic acid	$C_6H_{11}O_7^-$	1.28	195.0505	195.0508	-1.5	181.0715, 165.040	251,275
2	Syringaldehyde syringate or derivative of quinic acid	$C_{18}H_{17}O_9^-$	1.35	377.0873	377.0857	4.2	347.0745, 349.0905	267
3	Dihydroxy methoxy butanoic acid	$C_5H_9O_5^-$	1.39	149.0450	149.0449	0.6	131.0343, 119.0342, 135.0292, 103.0392	202, 241, 269
4	Syringaldehyde syringate or derivative of quinic acid	$C_{18}H_{17}O_9^-$	1.47	377.0878	377.0857	5.5	131.0342, 149.0449, 341.1090	267
5	Pentahydroxytetraoxoicosanoic acid	$C_{20}H_{31}O_{11}^-$	8.8	447.1866	447.1874	-1.7	437.1587, 401.1819, 199.1831, 155.1345	238
6	Tetrahydroxytetraoxoicosanoic acid	$C_{20}H_{31}O_{10}^-$	10.02	431.1917	431.1925	1.85	183.4840, 137.5310, 457.6654	239;276
7	Myricetin-3-O-hexoside	$C_{21}H_{19}O_{13}^-$	10.65	479.0826	479.0833	-0.003	125.0240, 287.0196	270; 354
8	Myricetin-3-arabinoside	$C_{20}H_{17}O_{12}^-$	10.82	449.0720	449.0728	-1.78	125.0235, 271.0246, 316.0229	272;344
9	Myrcetine rhamnose derivative	$C_{20}H_{17}O_{12}^-$	11.12	463.0877	463.0882	-1.07	151.0036, 178.9982, 271.0249, 316.0226, 317.0289 (aglycone myrcetin)	208, 262, 302
10	Quercetin-3-O-arabinoside	$C_{20}H_{17}O_{11}^-$	11.41	433.0771	433.0778	-1.61	151.0031, 178.9982 271.0246, 300.0287, 301.0343 (aglycone quercetin)	267, 353
11	Quercetin-3-O-pentoside	$C_{20}H_{17}O_{11}^-$	11.69	433.0771	443.0778	-1.61	151.0031, 178.9982, 271.024, 300.0269, 301.0346(aglycone quercetin),	269, 351
12	Quercetin 3-O-rhamnoside	$C_{21}H_{19}O_{11}^-$	11.70	447.0927	447.0934	-1.56	151.0030, 178.9982, 255.0298, 300.0298, 301.0347 (aglycone quercetin)	203, 257, 293, 349
13	Kaempferol-3-O-pentoside	$C_{20}H_{17}O_{10}^-$	12.17	417.0832	417.0822	2.39	255.0306, 285.0404 (aglycone kaempferol),	282

14	Kaempferol-3-O-rhamnoside	$C_{21}H_{19}O_{10}^-$	12.29	431.0978	431.0987	-2.08	255.0296, 285.0406 (aglycone kaempferol), 227.0345	269, 334
15	Diosmetin-6-c- glucoside or Hispidulin -7-glucoside	$C_{22}H_{21}O_{11}^-$	12.37	461.1084	461.1092	-1.73	431.0987, 283.0612	280
16	Luteolin	$C_{15}H_9O_6^-$	14.01	285.0399	285.0407	-2.80	133.0290	211, 284
17	Quercetin	$C_{15}H_9O_7^-$	14.13	301.0348	301.0356	-1.32	107.0130, 151.0029	211, 284
18	Oxilipin (Trihydroxyoctadecadienoic acid)	$C_{18}H_{31}O_5^-$	16.35	327.2171	327.2179	-2.75	211.3037	283
19	Apigenin	$C_{15}H_9O_5^-$	16.59	269.0450	269.0457	-2.60	107.0103, 117.0340	283
20	Chrysoeriol	$C_{16}H_{11}O_6^-$	17.24	299.0556	299.0563	-2.34	269.0455	283
21	Trihydroxyoctadecanoic acid	$C_{18}H_{33}O_5^-$	18.25	329.2328	329.2336	-2.43	183.1387	283
22	Dihydroxidodecadienoic acid	$C_{12}H_{19}O_4^-$	18.26	227.1283	227.1287	-1.76	245.0814, 183.1387, 171.0195, 115.1143, 183.1386, 277.8189	283
23	Unknown	$C_{28}H_{43}O_{11}^-$	20.67	555.2805	555.2846	7.38	-----	278
24	Unknown	$C_{28}H_{51}O_{13}N_9^-$	21.11	721.3606	721.3638	46.02	------	276
25	Unknown	$C_{30}H_{45}O_9N_7^-$	21.76	647.3337	647.3278	9.11	------	275
26	Hydroxyoctadecatrienoic acid	$C_{18}H_{29}O_3^-$	22.22	293.2117	293.2126	-3.06	------	275
27	Hydroxyoctadecatrienoic acid isomer	$C_{18}H_{29}O_3^-$	22.34	293.2117	293.2125	-2.72	------	275
28	Octahydroxyheptacosapentaenoic acid	$C_{27}H_{43}O_{10}^-$	22.60	527.2856	527.2864	-1.51	339.2002, 377.1429, 473.2830, 177.8446,151.3607, 335.1313,	275
29	Hydroxyoctadecadienoic acid	$C_{18}H_{31}O_3^-$	23.13	295.2279	295.2282	-1.01	------	275
30	Pentadecatetraenoic acid	$C_{15}H_{21}O_2^-$	23.35	233.1542	233.1546	-1.71	183.0118	276
31	Unknown	$C_{30}H_{41}O_{11}^-$	25.38	577.2649	577.2689	-6.92	------	275
32	Dihydroxypentadecatetraenoic acid	$C_{15}H_{21}O_4^-$	25.86	265.1440	265.1481	-15.46	------	275
33	Dihydroxypentadecatetraenoic acid Isomer	$C_{15}H_{21}O_4^-$	28.34	265.1440	265.1481	-15.46	------	275

* Daughter ions

Compounds associated with anthelmintic activity in Leucaena leucocephala

Glycosylated flavonoids. Nine glycosylated flavonoids corresponding to peaks 7, 8, 9, 10, 11, 12, 13, 14 and 15 were identified using UHPLC-Q/Orbitrap/MS/MS. Peak 7 with a [M−H]-ion at *m/z* 479.0833 and daughter ions at *m/z* 125.0240, 287.0196 were identified as myricetin-3-O-hexoside [13] Peak 8 with a [M-H]-ion *m/z* 449.0728 and daughter ions at *m/z* 271.0246, 125.0235 and 316.0229 were identified as myricetin-3-arabinoside [14]. Peak 9 with a [M−H]-ion 463.0882 and daughter ions at *m/z* 151.0036, 178.9982, 271.0249, 316.0226, 317.0289 (aglycone myricetin) were identified as myricetin rhamnose derivative [15], which was identified in *L. leucocephala* [16]. Peak 10 with a [M-H]-ion 433.0778 and daughter ions at *m/z* 151.0031, 178.9982, 271.0246, 300.0287 and 301.0343 (aglycone quercetin) were identified as quercetin-3-O-arabinoside, Aderogba and collaborators [17,18] identified in *L. leucocephala* quercetin-3-O-arabinofuranoside. Peak 11 with a [M-H]-ion 443.0778 and daughter ions at *m/z* 151.0031, 178.9982, 271.024, 300.0269, 301.0346 (aglycone quercetin moiety) were identified as quercetin-3-O-pentoside [19]. Peak 12 with a [M−H]-ion 447.0934 and daughter ions at *m/z* 151.0030, 178.9982, 255.0298, 300.0298 and 301.0347 (aglicone quercetin moiety) were identified as quercetin 3-O-rhamnoside [20], some authors [17,21,22] identified in *L. leucocephala* the same compound. Peak 13 with a [M−H]-ion 417.0882 and daughter ions at *m/z* 255.0306 and 285.0404 (aglycone kaempferol) were identified as kaempferol-3-O-pentoside [23]. Peak 14 with a [M-H]-ion 431.0987 and daughter ions at *m/z* 255.0296, 285.0406 (aglycone kaempferol) and 227.0345 were identified as kaempferol-3-O-rhamnoside. Peak 15 with a [M-H]-ion 461.1092 and daughter ions at *m/z* 431.0987 and 283.0612 were identified as diosmetin-6-c-glucoside or hispidulin-7-glucoside [24,25]*Flavonoids*. Four different flavonoids (peaks 16, 17, 19 and 20) were identified using UHPLC-Q/Orbitrap/MS/MS. Peak 16 with a [M-H]-ion 285.0407 and daughter ion at *m/z* 133.0290 were identified as luteolin [26], aditionally, luteolin has been identified in *L. leucocephala* [27]. Peak 17 with a [M-H]-ion 301.0356 and daughter ions at *m/z* 107.0130 and 151.0029 were identified as quercetin [24,28], compound that has also been identified in *L. leucocephala* [29]. Peak 19 with a [M-H]-ion 269.0457 and daughter ions at *m/z* 107.0103 and 117.0340 were identified as apigenin [30], compound identified in the same plant [17]. Peak 20 with a [M-H]-ion 299.0563 and daughter ion at *m/z* 269.0455 were identified as chrysoeriol [31], which was isolated from *L. leucocephala* [21](Hassan et al., 2014).

Methoxyphenols. Two methoxyphenols (peaks 2 and 4) were identified in the ethanolic extract using UHPLC-Q/Orbitrap/MS/MS. Peak 2 with a [M-H]-ion 377.0857 and daughter ions at *m/z* 347.0745 and 349.0905 and Peak 4 with a [M-H]-ion 377.0857 and daughter ions at *m/z* 131.0342, 149.0449 and 341.1090 were identified to as syringaldehyde syringate or derivative of quinic acid. Figure S3 shows the UHPLC total ion current chromatogram and Table S3 shows all the compounds detected and identified by UHPLC-Q/Orbitrap/MS/MS and in *Pitthecellobium dulce.*

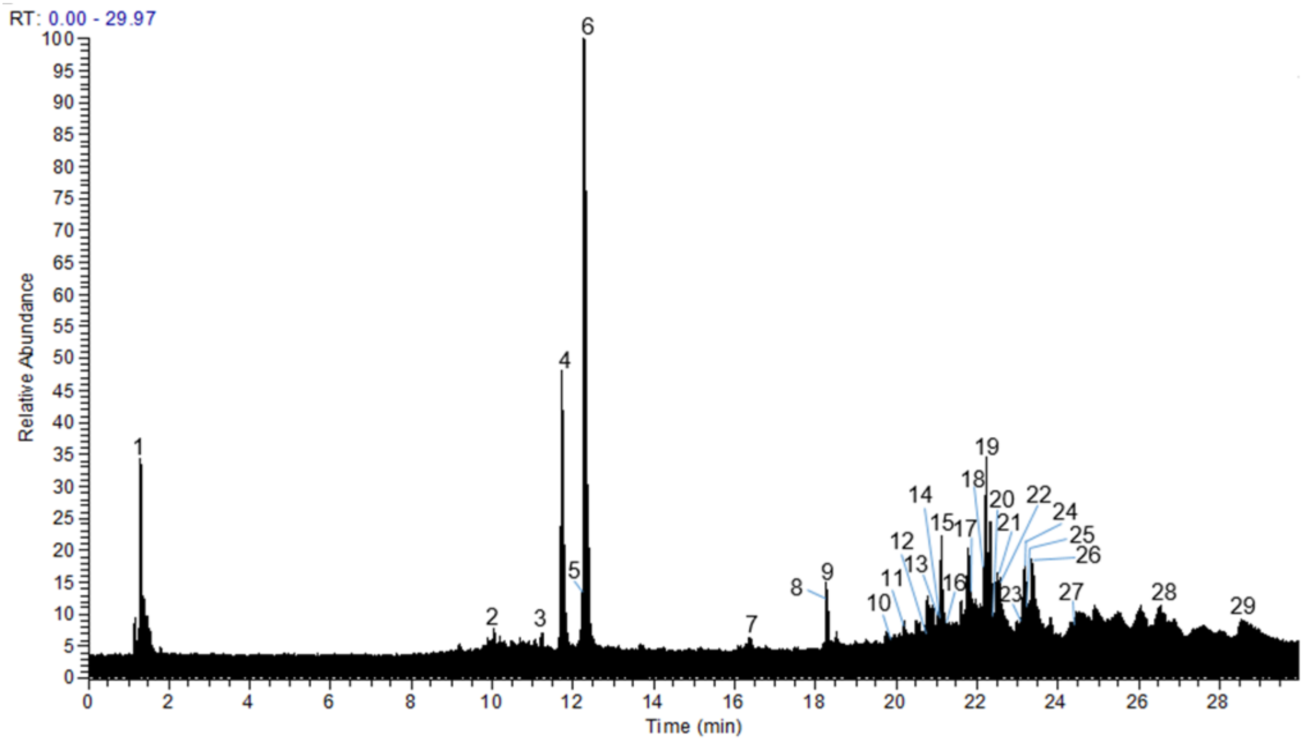

Figure S3. UHPLC TIC Chromatogram of *Pithecellobium dulce* ethanolic extract.

Table S3. Tentative identification of compounds in the ethanolic extract of *Pithecellobium dulce* by UHPLC-Q/Orbitrap/MS/ MS

Peak	Tentative identificaction	[M-H]$^-$ ions	Retention time (min.)	Theoretical mass (*m/z*)	Measured mass (*m/z*)	Accuracy (ppm)	Other ions (*m/z*)*	UV vis (nm)
1	Naphthalenedicarboxylic acid-O-hexose	$C_{18}H_{17}O_9^-$	1.31	377.0867	377.0859	-2.0	341.1093, 215.0326, 179.0556, 333.0962, 119.0343	278
2	Tetrahydroxytetraoxoicosanoic acid	$C_{20}H_{31}O_{10}^-$	10.03	431.1912	431.1927	3.5	--------	280
3	Quercetin-3-glucoside	$C_{21}H_{19}O_{12}^-$	11.22	463.0871	463.0887	3.3	301.0354 (quercetin aglycon), 300.0280, 179.0711, 151.0395	282
4	Luteolin-7-O-glucoside	$C_{21}H_{19}O_{11}^-$	11.74	447.0922	447.0937	3.2	151.003, 107.0132, 255.0294, 227.0345	264. 348
5	Azelaic acid	$C_9H_{15}O_4^-$	12.23	187.0965	187.0974	4.7	121.0654, 123.0808, 141.0914, 143.1073, 169.0866, 171.1020	282
6	Kaempferol-3-O-rhamnoside	$C_{21}H_{19}O_{10}^-$	12.29	431.0973	431.0988	3.6	255.0299, 285.0407 (kaempferol), 227.0348	229. 264. 290. 343
7	Trihydroxyoctadecadienoic acid	$C_{18}H_{31}O_5^-$	16.36	327.2166	327.2180	4.4	211.1338, 325.2023	283
8	Trihydroxyoctadecenoic acid	$C_{18}H_{33}O_5^-$	18.25	329.2323	329.2337	1.4	211.1337, 325.1847, 327.2181	283
9	Unknown	$C_{12}H_{19}O_4^-$	18.28	227.1278	227.1288	4.4	------	283
10	Dihydrocapsiate	$C_{18}H_{27}O_4^-$	19.75	307.1904	307.1919	4.9	121.0653, 235.1340, 211.1339, 185.1180, 151.0395	282
11	Hydroxydioxoheptadecenoic acid	$C_{17}H_{27}O_5^-$	20.20	311.1853	311.1865	3.8	171.1022, 267.1968	281
12	Phenethyl butyrate	$C_{12}H_{15}O_2^-$	20.78	191.1067	191.1077	5.2	-----	278

13	Hydroperoxyoctadecatrienoic acid	$C_{18}H_{29}O_4^-$	20.90	309.2060	309.2075	4.6	-------	278
14	Methoxy heptaetilenglicol	$C_{15}H_{31}O_8^-$	20.98	339.2019	339.2005	4.1		278
15	Unknown	$C_{30}H_{53}O_{14}N_6$	21.12	721.3620	721.3641	-2.9	---------	
16	Methoxy heptaetilenglicol isomer	$C_{15}H_{31}O_8^-$	21.23	339.2019	339.2005	4.1	309.2075, 325.1848	278
17	Unknown	$C_{28}H_{43}O_{11}^-$	21.82	555.2805	555.2847	-7.5	--------	275
18	Farrerol derivative	$C_{29}H_{37}O_6^-$	22.13	481.2585	481.2578	-1.3	151.0396	275
19	Hydroxyoctadecatrienoic acid	$C_{18}H_{29}O_3^-$	22.22	293.2111	293.2126	4.9	275.2018, 195.1386	275
20	Hydroxyoctadecatrienoic acid isomer	$C_{18}H_{29}O_3^-$	22.33	293.2111	293.2126	5.2	277.2173, 275.2018, 195.1387	275
21	Oxo-octadecatrienoic acid	$C_{18}H_{27}O_3^-$	22.47	291.1955	291.1971	5.4	277.2176, 275.2023, 195.1387	275
22	Oxo-octadecatrienoic acid isomer	$C_{18}H_{27}O_3^-$	22.57	291.196	291.1971	-3.7	277.2176, 275.2023, 195.1387	
23	Heptaetilenglicol	$C_{14}H_{29}O_8^-$	23.09	325.1862	325.1848	4.3	309.1744, 251.1481	275
24	Hydroxyoctadecadienoic acid	$C_{18}H_{31}O_3^-$	23.18	295.2268	295.2283	5.2	277.2175, 195.1388, 279.1964, 253.1805	275
25	Heptaetilenglicol isomer	$C_{14}H_{29}O_8^-$	23.28	325.1862	325.1848	4.3	309.1747, 251.1483	275
26	Pentadecatetraenoic acid	$C_{15}H_{21}O_2^-$	23.36	233.1536	233.1547	4.7	------	275
27	Heptaetilenglicol isomer	$C_{14}H_{29}O_8^-$	24.80	325.1862	325.1848	4.3	309.1743, 251.1485	
28	Unknown	$C_{27}H_{43}O_{14}^-$	26.55	591.2653	591.2614	6.5	--------	
29	Unknown	$C_{22}H_{25}O_2^-$	26.86	321.1860	321.1825	9.3	-----------	

* Daughter ions

147

148

149

Compounds associated with anthelmintic activity in Pithecellobium dulce

Glycosylated flavonoids. Four glycosylated flavonoids corresponding to peaks 3, 4, 6 and 18 were identified using UHPLC-Q/Orbitrap/MS/MS. Peak 3 with a [M-H]-ion *m/z* 463.0887 and daughter ions at *m/z* 301.0354 (aglycone quercetin) 300.0280, 179.0711 and 151.0395 were identified as quercetin-3-glucoside [3,32]. Peak 4 with a [M−H]-ion *m/z* 447.0937 and daughter ions at *m/z* 151.003, 107.0132, 255.0294 and 227.0345 were identified as luteolin-7-O-glucoside [33]. Peak 6 with a [M−H]-ion *m/z* 431.0988 and daughter ions at *m/z* 255.0299, 285.0407 and 227.0348 were identified as kaempferol-3-O-rhamnoside [34], previously, this compound had been identified in *P. dulce* [35].

Methoxyphenols. One phenol was identified using UHPLC-Q/Orbitrap/MS/MS. Peak 10 with a [M−H]-ion *m/z* 307.1919 and daughter ions at *m/z* 121.0653, 235.1340, 211.1339, 185.1180 and 151.0395 were identified as dihydrocapsiate [36].

Fatty acids. One fatty acid was identified using UHPLC-Q/Orbitrap/MS/MS. Peak 5 with a [M−H]-ion *m/z* 187.0974 and daughter ions at *m/z* 121.0654. 123.0808. 141.0914. 143.1073. 169.0866 and 171.1020 was identified as azelaic acid [12].

References

1. Makita, C., Chimuka, L., Steenkamp, P., Cukrowska, E., & Madala, E. Comparative analyses of flavonoid content in *Moringa oleifera* and *Moringa ovalifolia* with the aid of UHPLC-qTOF-MS fingerprinting. *S. Afr. J. Bot*. **2016**, 105, 116–122. [CrossRef]
2. Simirgiotis, M.J., Quispe, C., Areche, C., & Sepulveda, B. Phenolic Compounds in Chilean Mistletoe (Quintral, *Tristerix tetrandus*) Analyzed by UHPLC-Q/Orbitrap/MS/MS and Its Antioxidant Properties. *Molecules*, **2016** 21(3), 245. [CrossRef] [PubMed]
3. Karar, M. G. E., & Kuhnert, N. UPLC-ESI-Q-TOF-MS/MS characterization of phenolics from *Crataegus monogyna* and *Crataegus laevigata* (Hawthorn) leaves, fruits and their herbal derived drops (Crataegutt Tropfen). J. *Chem. Biol. Ther*. **2015,** 1, 1–23 [CrossRef]
4. Simirgiotis, M.J., Quispe, C., Mocan, A., Villatoro, J. M., Areche, C., Bórquez, J., Sepúlveda, B., Echiburu-Chau, C. UHPLC high resolution orbitrap metabolomic fingerprinting of the unique species *Ophryosporus triangularis* Meyen from the Atacama Desert, Northern Chile. *Rev. Bras. Farmacogn*. **2017**, 27(2), 179–187. [CrossRef]
5. Abu-Reidah, I. M., Arráez-Román, D., Segura-Carretero, A., & Fernández-Gutiérrez, A. Extensive characterisation of bioactive phenolic constituents from globe artichoke (*Cynara scolymus* L.) by HPLC–DAD-ESI-QTOF-MS. *Food. Chem.* **2013,** *141*(3), 2269-2277. [CrossRef]
6. Choi, J., An, X., Lee, B. H., Lee, J. S., Heo, H. J., Kim, T., Ahn, J.W., Kim, D.O. Protective effects of bioactive phenolics from jujube (*Ziziphus jujuba*) seeds against H2O2–induced oxidative stress in neuronal PC-12 cells. *Food. Sci. Biotechnol*. **2015**, 24(6), 2219–2227. [CrossRef]
7. Kumar, S., Chandra, P., Bajpai, V., Singh, A., Srivastava, M., Mishra, D. K., & Kumar, B. Rapid qualitative and quantitative analysis of bioactive compounds from *Phyllanthus amarus* using LC/MS/MS techniques. *Ind. Crops. Prod*. **2015**, 69, 143-152. [CrossRef]
8. Xu, M., Shao, Q., Ye, S., Li, S., Wu, M., Ding, M., & Li, Y. Simultaneous extraction and identification of phenolic compounds in *Anoectochilus roxburghii* using microwave-assisted extraction combined with UPLC-Q-TOF-MS/MS and their antioxidant activities. *Front. Plant. Sci*. **2017**, 8, 1474. [CrossRef] [PubMed]
9. Ramamoorthy, M., & Paliwal, K. Allelopathic compounds in leaves of *Gliricidia sepium* (Jacq.) kunth ex walp. and its effect on *Sorghum vulgare L. J. Chem. Ecol*. **1993**, 19(8), 1691–1701. [CrossRef]

10. Abu-Reidah, Ali-Shtayeh, M. S., Jamous, R. M., Arráez-Román, D., & Segura-Carretero, A. Comprehensive metabolite profiling of *Arum palaestinum* (Araceae) leaves by using liquid chromatography–tandem mass spectrometry. *Food. Res. Int*. **2015**, 70, 74–86. [CrossRef]
11. Fu, J., Wang, M., Guo, H., Tian, Y., Zhang, Z., & Song, R. Profiling of components of rhizoma et radix polygoni cuspidati by high-performance liquid chromatography with ultraviolet diode-array detector and ion trap/time-of-flight mass spectrometric detection. *Pharmacogn. Mag*. **2015**, 11(43), 486–501. [CrossRef]
12. Ma, X.Q., Leung, A.K.M., Chan, C. L., Su, T., Li, W.D., Li, S. M., Fong, D.W.F., Yu, Z. L. UHPLC UHD Q-TOF MS/MS analysis of the impact of sulfur fumigation on the chemical profile of Codonopsis Radix (Dangshen). *Analyst*. **2014**, 139(2), 505–516. [CrossRef]
13. Hofmann, T., Nebehaj, E., & Albert, L. Antioxidant properties and detailed polyphenol profiling of European hornbeam (*Carpinus betulus L*.) leaves by multiple antioxidant capacity assays and high-performance liquid chromatography/multistage electrospray mass spectrometry. *Ind. Crops. Prod*. **2016**, 87, 340–349. [CrossRef]
14. Sójka, M., Guyot, S., Kołodziejczyk, K., Król, B., & Baron, A. Composition and properties of purified phenolics preparations obtained from an extract of industrial blackcurrant (*Ribes nigrum L*.) pomace. *J. Hortic. Sci. Biotechnol.* **2009**, 84(6), 100–106. [CrossRef]
15. Bystrom, L. M., Lewis, B. A., Brown, D. L., Rodriguez, E., & Obendorf, R. L. Characterization of phenolics by LC-UV/vis, LC-MS/MS and sugars by GC in *Melicoccus bijugatus* Jacq. "Montgomery" fruits. *Food. Chem*. **2008**, 111(4), 1017–1024. [CrossRef] [PubMed]
16. Lowry, J. B., Cook, N., & Wilson, R. D. Flavonol glycoside distribution in cultivars and hybrids of *Leucaena leucocephala*. *J. Sci. Food. Agric*. **1984**, 35(4), 401–407. [CrossRef]
17. Aderogba, M. A., McGaw, L. J., Bezabih, B. T., & Abegaz, B. M. Antioxidant activity and cytotoxicity study of *Leucaena leucocephala* (Lam.) de wit leaf extract constituents. Niger *J. Nat. Prod. Med.* **2010**, 13(1), 65–68. [CrossRef]
18. Borges, G., Degeneve, A., Mullen, W., & Crozier, A. Identification of flavonoid and phenolic antioxidants in black currants, blueberries, raspberries, red currants, and cranberries. *J. Agric. Food Chem*. **2010**, 58(7), 3901–3909. [CrossRef]
19. Engels, C., Gräter, D., Esquivel, P., Jiménez, V. M., Gänzle, M. G., & Schieber, A. Characterization of phenolic compounds in jocote (*Spondias purpurea L*.) peels by ultra high-performance liquid chromatography/electrospray ionization mass spectrometry. *Food. Res. Int*. **2012**, 46(2), 557–562. [CrossRef]

20. Li, A., Hou, X., & Wei, Y. Fast screening of flavonoids from switchgrass and *Mikania micrantha* by liquid chromatography hybrid-ion trap time-of-flight mass spectrometry. *Anal. Methods*. **2018**, 10(1), 109–122. [CrossRef]
21. Hassan, R. A., Tawfik, W. A., & Abou-Setta, L. M. The flavonoid constituents of *Leucaena leucocephala*. Growing in Egypt, and their biological activity. *Afr. J. Tradit. Complement. Altern. Med.* **2014**, 11(1), 67–72. [CrossRef] [PubMed]
22. Negi, P., Rawat, B. S., & Negi, D. S. Antifeedant Constituents from *Leucaena leucocephala*. *J. Appl. Pharm. Sci*. **2016**, 6(12), 28–31. [CrossRef]
23. Li, Z. H., Guo, H., Xu, W. B., Ge, J., Li, X., Alimu, M., & He, D. J. Rapid identification of flavonoid constituents directly from PTP1B inhibitive extract of raspberry (*Rubus idaeus L.*) leaves by HPLC–ESI–QTOF–MS-MS. *J. Chromatogr. Sci.* **2016**, 54(5), 805-810. [CrossRef] [PubMed]
24. Brito, A., Ramirez, J. E., Areche, C., Sepulveda, B., & Simirgiotis, M. J. HPLC-UV-MS profiles of phenolic compounds and antioxidant activity of fruits from three citrus species consumed in Northern Chile. *Molecules*. **2014**, 19(11), 17400–17421. [CrossRef]
25. Mena, P., Cirlini, M., Tassotti, M., Herrlinger, K. A., Dall'Asta, C., & Del Rio, D. Phytochemical Profiling of Flavonoids, Phenolic Acids, Terpenoids, and Volatile Fraction of a Rosemary (*Rosmarinus officinalis L.*) Extract. *Molecules*. **2016**, 21(11). [CrossRef]
26. McNab, H., Ferreira, E. S. B., Hulme, A. N., & Quye, A. Negative ion ESI–MS analysis of natural yellow dye flavonoids—An isotopic labelling study. *Int. J. Mass. Spectrom*. **2009**, 284(1), 57–65. [CrossRef]
27. Zarina, Z., Ruzaidi, C. M., Sam, S. T., & Mustafa Al Bakri, A. M. Investigation on Antioxidants Compounds Composition Contains in *Leucaena Leucocephala* (Petai Belalang). *IOP. Conf. Ser. Mater. Sci. Eng*. **2019**, 551, 12016. [CrossRef]
28. Saldanha, L. L., Vilegas, W., & Dokkedal, A. L. Characterization of flavonoids and phenolic acids in *Myrcia bella* Cambess. using FIA-ESI-IT-MS(n) and HPLC-PAD-ESI-IT-MS combined with NMR. *Molecules*. **2013**, 18(7), 8402–8416. [CrossRef] [PubMed]
29. Abdelhady, N. M., & Abdallah, G. M. HPLC/MS/MS study of phenolic compounds of *Leucaena leucocephala* legumes monitored with their *in vitro* antihyperglycemic activity. *European. J. Med. Plants*. **2016**, 17(4), 1–9. [CrossRef]
30. Fu, S., Segura-Carretero, A., Arraez-Roman, D., Menendez, J. A., De La Torre, A., & Fernandez-Gutierrez, A. Tentative characterization of novel phenolic compounds in extra

virgin olive oils by rapid-resolution liquid chromatography coupled with mass spectrometry. *J. Agric. Food. Chem*. **2009**, 57(23), 11140–11147. [CrossRef]
31. Liang, S., Xiong, Z., Tian, J., & Zhang, W.D. Flavones from Daphne feddei. *Chem. Nat. Compd*. **2011**, 47(5), 816–817. [CrossRef]
32. Bell, L., Oruna-Concha, M. J., & Wagstaff, C. Identification and quantification of glucosinolate and flavonol compounds in rocket salad (*Eruca sativa*, *Eruca vesicaria* and *Diplotaxis tenuifolia*) by LC-MS: highlighting the potential for improving nutritional value of rocket crops. *Food. Chem*. **2015**, 172, 852–861. [CrossRef] [PubMed]
33. Ibrahim, R. M., El-Halawany, A. M., Saleh, D. O., El Naggar, E. M. B., El-Shabrawy, A. E. R. O., & El-Hawary, S. S. HPLC-DAD-MS/MS profiling of phenolics from *Securigera securidaca* flowers and its anti-hyperglycemic and anti-hyperlipidemic activities. *Rev. Bras. Farmacogn*. **2015**, *25*(2), 134-141. [CrossRef]
34. Neugart, S., Rohn, S., & Schreiner, M. Identification of complex, naturally occurring flavonoid glycosides in *Vicia faba* and *Pisum sativum* leaves by HPLC-DAD-ESI-MSn and the genotypic effect on their flavonoid profile. *Food. Res. Int*. **2015**, 76, 114–121. [CrossRef]
35. Nigam, S. K., & Mitra, C. R. *Pithecolobium dulce*. V. Chemistry of the seed saponin and constituents of the leaves. *Planta. Med*. **1970**, 18(1), 44–50. [CrossRef]
36. Singh, S., Jarret, R., Russo, V., Majetich, G., Shimkus, J., Bushway, R., & Perkins, B. Determination of capsinoids by HPLC-DAD in *capsicum* species. *J. Agric. Food. Chem*. **2009**, 57(9), 3452–3457. [CrossRef]
